# Draft Genome Sequences of *Burkholderia cenocepacia* ET12 Lineage Strains K56-2 and BC7

**DOI:** 10.1128/genomeA.00841-13

**Published:** 2013-10-17

**Authors:** John J. Varga, Liliana Losada, Adrian M. Zelazny, Maria Kim, Jamison McCorrison, Lauren Brinkac, Elizabeth P. Sampaio, David E. Greenberg, Indresh Singh, Cheryl Heiner, Meredith Ashby, William C. Nierman, Steven M. Holland, Joanna B. Goldberg

**Affiliations:** Department of Microbiology, Immunology, and Cancer Biology, University of Virginia Health System, Charlottesville, Virginia, USAa; Department of Pediatrics and the Center for Cystic Fibrosis Research, Emory University School of Medicine, Children’s Healthcare of Atlanta, Inc., Atlanta, Georgia, USAb; The J. Craig Venter Institute, Rockville, Maryland, USAc; Microbiology Service, Department of Laboratory Medicine, Clinical Center, National Institutes of Health, Bethesda, Maryland, USAd; Laboratory of Clinical Infectious Diseases, National Institute of Allergy and Infectious Diseases, National Institutes of Health, Bethesda, Maryland, USAe; University of Texas Southwestern Medical School, Dallas, Texas, USAf; Pacific Biosciences, Menlo Park, California, USAg; The George Washington University, Washington, DC, USAh

## Abstract

The *Burkholderia cepacia* complex (BCC) is a group of closely related bacteria that are responsible for respiratory infections in immunocompromised humans, most notably those with cystic fibrosis (CF). We report the genome sequences for *Burkholderia cenocepacia* ET12 lineage CF isolates K56-2 and BC7.

## GENOME ANNOUNCEMENT

The *Burkholderia cepacia* complex (BCC) consists of 17 genetically related but phenotypically distinct betaproteobacterial species. BCC species can be found in a variety of niches and are capable of infecting numerous hosts. The most well-known member of the BCC is *Burkholderia cenocepacia*, a pathogen of onions and immunocompromised humans (1). We report the genome sequences of *B. cenocepacia* strains BC7 (2) and K56-2 (3), members of the highly transmissible genomovar III ET12 lineage (4, 5). While a genome sequence exists for the ET12 lineage *B. cenocepacia* strain J2315 (6), BC7 was isolated from a patient with “cepacia syndrome” and K56-2 is less antibiotic resistant, making it more amenable to genetic manipulation.

Genomic DNA was prepared with the DNeasy blood and tissue kit (Qiagen), according to the manufacturer’s instructions. A combination of the Roche 454 GS-FLX Titanium 8-kb mate-pair libraries (~12× coverage) and 100-bp Illumina fragment reads (50× coverage) were used for sequence determinations. All reads were used to generate hybrid assemblies with Celera Assembler version 7.0 (CA7.0) (7). Almost all of the intrascaffold breaks in these assemblies were estimated at <20 bases, suggesting that they are ambiguities likely resulting from the high content of short tandem repeats in the G+C-rich regions of the genome. Thus, we sought additional PacBio reads to obtain a better consensus of the K56-2 genome; short- and long-length PacBio reads (<2,000 and >2,000, respectively) were used to generate highly accurate, long, preassembled reads using the HGAP algorithm (Pacific Biosciences), which were combined with 454 reads and input into the CA7.0. K56-2 assembled into 14 scaffolds containing 19 contigs. The final contig sequences were then run through the consensus polisher program, Quiver (PacBio), resulting in 3,516 base changes and 624 base insertions or deletions.

The BC7 assembly was initially in 7 scaffolds and 785 contigs. Since additional sequencing was not pursued for this genome, a reference-guided gap closure pipeline was employed to resolve hundreds of gaps found throughout the assembly. The gap sequences were predicted from the closed-genome reference (J2315) and used to recruit and locally assemble reads into the gaps to merge the adjacent contigs. The resulting assembly is 296 contigs in 7 scaffolds.

As in J2315, each genome has 3 chromosomes and 1 plasmid (6). The chromosomes in BC7 and K56-2 have very similar sizes to those reported in J2315 (3.83 Mb, 3.19 Mb, and 0.88 Mb), except for chromosome 1 in K56-2, which has an estimated size of 3.67 Mb, due to the absence of the large duplication in J2315. The presence of the plasmid was previously detected in K56-2, BC7, and J2315 (8). Sequence data confirm the presence of the plasmid in BC7 and K56-2, with practically no differences between the three strains except for the presence of an additional copy of an insertion element in the J2315 plasmid, pBCJ2315 (6). The genomes were annotated using the annotation pipeline of the J. Craig Venter Institute (JCVI) (http://www.jcvi.org) and submitted to GenBank. Sequence data indicate that K56-2 and BC7 have similar gene contents to that of J2315, with 7,714 and 7,930 open reading frames (ORFs), respectively.

### Nucleotide sequence accession numbers.

The *B. cenocepacia* BC7 whole-genome shotgun project has been deposited at DDBJ/EMBL/GenBank under the accession no. ALIZ00000000. The version described in this paper is the second version, ALIZ01000000.

The *B. cenocepacia* K56-2 whole-genome shotgun project has been deposited at DDBJ/EMBL/GenBank under the accession no. ALJA00000000. The version described in this paper is the second version, ALJA01000000.
